# Relationship Between Dietary Choline Intake and Cognitive Function in the United States: A Cross‐Sectional Study of the 2011–2014 NHANES Cycle

**DOI:** 10.1002/fsn3.71631

**Published:** 2026-03-11

**Authors:** Yu‐hang Chen, Ting‐ting Liu, Li‐ming Chen

**Affiliations:** ^1^ Department of Operations Management Chongqing Mental Health Center Chongqing Chongqing China; ^2^ Department of Clinical Nutrition Chongqing Mental Health Center Chongqing Chongqing China; ^3^ Chongqing Mental Health Center Chongqing Chongqing China

**Keywords:** choline, cognitive function, dietary, elderly, NHANES, regression analysis

## Abstract

**Background:**

Current evidence on the correlation between dietary choline intake and Cognitive function is inconsistent. Therefore, this study examines the relationship between dietary choline intake and Cognitive function among noninstitutionalized older adults in the United States based on individuals in the National Health and Nutrition Examination Survey (NHANES) database.

**Methods:**

This cross‐sectional analysis used data from the NHANES conducted from 2011 to 2014. Two 24‐h dietary recalls were used to measure choline intake, which was then weighted. The Consortium to Establish a Registry for Alzheimer's Disease (CERAD) test, the Digit Symbol Substitution Test (DSST), and the Animal Fluency Test (AFT) were used to evaluate cognitive function. People were considered to have low cognitive function if their scores on each cognitive test fell below the lowest quartile for their respective age group. The association between choline intake and cognitive function was evaluated using restricted cubic splines (RCS) and binary logistic regression.

**Results:**

In the fully corrected model, the odds ratio (OR) and 95% confidence intervals (CI) of CERAD‐WL test scores, CERAD‐DR test scores, AFT scores, and DSST scores for the highest quartile of dietary choline intake were 0.61 (0.46 ~ 0.81), 0.66 (0.51 ~ 0.85), 0.66 (0.50 ~ 0.88), and 0.58 (0.42 ~ 0.80); the OR and 95% CI of CERAD‐WL test scores, CERAD‐DR test scores, AFT scores, and DSST scores for the highest quartile of total choline intake were 0.62 (0.47 ~ 0.83), 0.68 (0.53 ~ 0.88), 0.65 (0.49 ~ 0.86), and 0.57 (0.42 ~ 0.79). Subgroup analysis suggests that the interaction between gender, hypertension status, and choline intake has some impact on the relationship model. In dose–response relationship studies, dietary choline intake and total choline intake are both *U*‐shaped correlated with DSST scores.

**Conclusion:**

Choline intake can improve cognitive function scores, and a nonlinear relationship exists between the two.

## Graphical abstract

In this study, we found a negative relationship between choline intake (dietary choline and total choline) and cognitive function in the elderly population of the United States. RCS analysis showed a *U*‐shaped association between choline intake and cognitive test (DSST) scores. Interaction analysis suggests that gender and hypertension status may affect the association between choline intake and cognitive function.

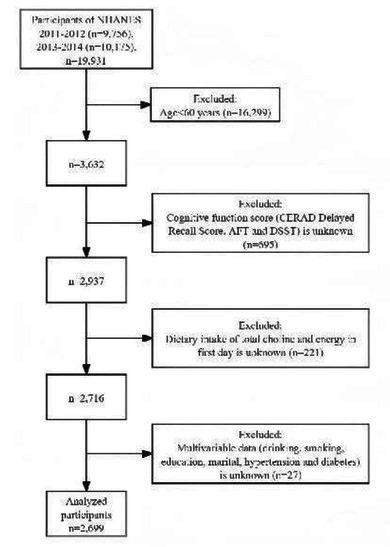

## 1 Introduction

The global demographic structure is changing rapidly as the global population is aging. The number of elderly people aged 65 and above has grown rapidly from 461 million in 2004, and is expected to reach approximately 1.5 billion by 2050, accounting for over 17% of the world's total population (Brivio et al. 2019). According to a previous study in the United States, nearly one‐third of people aged 65 years and older suffer from dementia or mild cognitive impairment (Manly et al. 2022). The global incidence of dementia is predicted to increase from 57.4 million cases in 2019 to 152.8 million cases in 2050 (Estimation of the Global Prevalence of Dementia in 2019 and Forecasted Prevalence in 2050: An Analysis for the Global Burden of Disease Study 2019 2022), and the costs of dementia‐related healthcare will exceed 800 billion dollars in 2021 alone (Montero‐Odasso et al. 2023). This indicates that Alzheimer's disease (AD) and related dementia present a significant challenge to the global healthcare system, making it crucial from a social and medical standpoint to prioritize the cognitive health of the elderly population.

Choline is an essential nutrient with multiple biological functions, closely associated with healthy brain function. It serves as a methyl group donor and an alternative pathway for methionine synthesis, with methionine acting as a precursor for phospholipid synthesis. Phospholipids are crucial for maintaining cell membrane integrity and facilitating intracellular signaling (Institute of Medicine Standing Committee on the Scientific Evaluation of Dietary Reference I, its Panel on Folate OBV, Choline 1998; Zeisel and Blusztajn 1994). Additionally, choline is a precursor for acetylcholine synthesis, a neurotransmitter involved in the cholinergic neural network associated with memory (Bowen et al. 1976; Institute of Medicine Standing Committee on the Scientific Evaluation of Dietary Reference I, its Panel on Folate OBV, Choline 1998; Zeisel and Blusztajn 1994). The loss of brain cell membrane function and intercellular communication is a hallmark of AD (Azam et al. 2021). Fortunately, choline is relatively easy to obtain. On one hand, the human body can synthesize small amounts of choline through the liver's phosphatidylethanolamine N‐methyltransferase pathway. On the other hand, increasing dietary choline intake is the primary method for preventing choline deficiency (Arias et al. 2020). Therefore, researchers have found that dietary choline exerts neuroprotective and memory‐preserving effects (Blusztajn et al. 2017), and increasing choline intake may slow the progression of AD (Higgins and Flicker 2003).

However, epidemiological studies on choline intake and cognitive function are limited, yielding mixed results. In an observational study that included 247 dementia cases, it was demonstrated that lower dementia risk and Alzheimer's disease were each associated with increased dietary choline intake (Eslami et al. 2024). In another study evaluating the association between choline supplementation and dementia risk in middle‐aged and older men, it was demonstrated that higher total choline intake was not associated with dementia risk events (Ylilauri et al. 2019). There have also been some observational studies investigating the relationship between dietary choline and cognitive function in recent years, but the results have varied. In two recent studies using the NHANES database, An et al. (An et al. 2023) found that dietary (OR = 0.94, 95% CI = 0.75 ~ 1.17) and total choline intake (OR = 0.87, 95% CI = 0.70 ~ 1.09) were not associated with changes in cognitive test scores. Liu et al.'s study (Liu et al. 2021) showed that a certain dose of total choline intake can reduce the risk of cognitive impairment by about 50%.

Although most previous studies have indicated that dietary choline intake correlates with better cognitive performance, the specific form of its dose–response relationship remains unclear. For individuals with insufficient intake, increasing choline consumption may yield significant cognitive improvements. However, when intake reaches or exceeds physiological requirements, marginal benefits may diminish. This potential nonlinear relationship was also noted in a prospective cohort study (Yuan et al. 2022). Therefore, assuming a purely linear relationship may not fully capture the actual association between choline intake and cognitive function.

To comprehensively investigate potential research gaps regarding the nonlinear relationship between choline intake and cognitive function, we conducted a secondary data analysis of the U.S. population using the NHANES database, incorporating a RCS model. This study also provides valuable insights for future studies on choline metabolism and the potential mechanisms underlying the development of cognitive impairment or AD.

## 2 Methods

### 2.1 Data Source and Study Population

**FIGURE 1:**
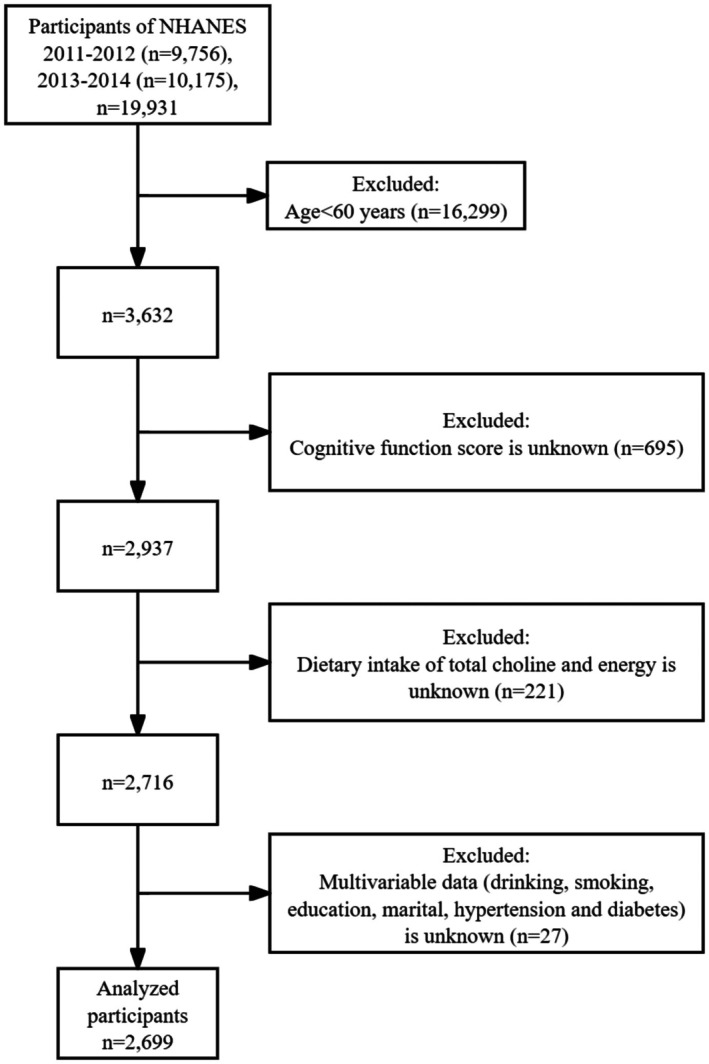
Sample selection flowchart for National Health and Nutrition Examination Survey 2011–2014.

It is a secondary analysis of NHANES database, utilizing data from two cycles conducted between 2011 and 2014, employing a stratified multistage probability sampling design to ensure that the sampled population is representative of the noninstitutionalized civilian population in the United States (Lu and Ni 2023). Data for this study included information from structured household interviews and physical assessments conducted at mobile examination centers (MECs) to provide a comprehensive assessment of the health and nutritional status of the U.S. population, primarily through interviews, physical examinations, and laboratory tests (Wang et al. 2023). NHANES was approved by the National Center for Health Statistics (NCHS) Ethics Review Board, and before enrollment, a written informed consent form was obtained from participants aged 18 years and above. The study followed the STROBE reporting guidelines to enhance the reporting of observational epidemiologic studies. As the analyses involved secondary data, they did not fall into the category of “human subjects research” and therefore did not require further approval from the Institutional Review Board of the Centers for Disease Control and Prevention (CDC) (Zhang et al. 2023). For more information, please visit https://www.cdc.gov/nchs/nhanes/. 2699 participants aged ≥ 60 years were included in this study, and the sample selection flowchart is shown in Figure 1.

### 2.2 Dietary Choline Intake Assessment

Based on the Food and Nutrient Database for Food Studies supplied by the United States Department of Agriculture (USDA), two 24‐h food recall interviews were used to measure dietary choline intake. Following the initial in‐person dietary interview at a MEC, follow‐up dietary interviews were completed over the phone from the home office. On the second occasion, the interviewer would arrange for the subject to have a telephone follow‐up interview 3–10 days after the MEC dietary interview (Jieru et al. 2024; Jun et al. 2021). Participants self‐reported the kind and quantity of all dietary supplements they took for two 24‐h dietary recalls, which provided information on supplemental choline intake.

Dietary and supplementary choline intakes were included in the actual total choline intake in this investigation. During the same 24‐h recall period, dietary choline and total choline from food and supplements were examined independently. Total choline consumption was calculated by averaging the total choline from supplements and food choline during the two recall days (Li et al. 2022). Weight selection was based on the following criteria: The first dietary recall weight (WTDRD 1) was utilized when there was no second dietary recall weight; if there was a second dietary recall weight (including whether a first dietary recall weight was present or not), the second dietary recall weight (WTDRD 2) was used (Shi et al. 2024).

### 2.3 Cognitive Function Assessment

According to the NHANES website (https://wwwn.cdc.gov/Nchs/Nhanes/2013‐2014/CFQ_H.htm), participants' cognitive function was evaluated using the NHANES 2011–2014 Cognitive Function Module. Participants 60 years of age and older were given cognitive tests. At a mobile screening facility, assessments were performed in person by qualified interviewers. To assess participants' cognitive function, the following tests of cognitive function were used, with higher scores indicating better cognitive function. The CERAD test consists of three consecutive learning trials (CERAD‐WL) and one delayed recall trial (CERAD‐DR) designed to assess immediate and delayed recall of new verbal information, so results are shown as three separate trial scores ranging from 0 to 10, a total score ranging from 0 to 30 for all three trials, and a delayed recall score ranging from 0 to 10 (Morris et al. 1989). With scores ranging from 3 to 40, the AFT evaluates category verbal fluency (Clark et al. 2009). With scores ranging from 0 to 105, the DSST, a part of the Wechsler Adult Intelligence Scale, assesses working memory, sustained attention, and processing speed (Salthouse 1992). Currently, there is no gold standard for the CERAD test, AFT, and DSST to diagnose low cognitive function. Therefore, the threshold for this study was defined based on the lowest quartile (25th percentile) (Chen et al. 2017).

### 2.4 Covariates

For this study, potential covariates and confounders were identified, including gender, age, race, education, marital status, poverty/income ratio (PIR), body mass index (BMI), smoking status, alcohol intake status, diagnosis of diabetes mellitus, diagnosis of hypertension, and total dietary energy intake. At screening, older participants were categorized as 60–69 years, 70–79 years, and ≥ 80 years. Gender was categorized as male or female. Race categorizes people based on the composition of the U.S. population, including Mexican American, other Hispanic, Non‐Hispanic White, Non‐Hispanic Black, and other races (Ji et al. 2024). Educational attainment was categorized into 3 levels: high school or less, some college, and college graduate or higher. Marital status was categorized into 3 groups: married/living with a partner, widowed/divorced/separated, and never married. PIR was calculated by dividing household (or individual) income by a specific poverty criterion for the year of the survey and are used as a measure of socio‐economic status (Minhas et al. 2023). BMI was categorized as Underweight (< 18.5 kg/m2), Normal (18.5 to < 25) BMI was categorized as Underweight (< 18.5 kg/m2), Normal (18.5 to < 25 kg/m^2^), Overweight (25 to < 30 kg/m2) and Obese (30 kg/m^2^ or greater). Smoking status was categorized into 3 groups: never smokers, former smokers, and current smokers. Alcohol intake status was categorized into 4 groups: Non‐drinker, 1 to < 5 drinks/month, 5 to < 10 drinks/month, and 10 drinks/month or greater (Christensen et al. 2020). Hypertension and diabetes mellitus were defined as self‐reported physician‐diagnosed hypertension and diabetes mellitus (Dong et al. 2020). Total energy intake data were obtained from the 24‐h dietary recall survey in the NHANES database and processed in the same manner as dietary choline intake data (Xu et al. 2024).

Among the covariates we adjusted for, three contained missing data, with data completeness rates of 92.4% for PIR, 99.2% for alcohol drinking, and 98.7% for BMI. Considering that analyzing only the complete data would result in a loss of sample size and may introduce selection bias, we employed multiple imputations to address missing data for all covariates in the regression models. Multiple interpolation provides more efficient and less biased estimates by creating multiple complete datasets that reflect the uncertainty of the missing values (Lu et al. 2025).

### 2.5 Statistical Analysis

All analyses were conducted using R (version 4.4.1) and took into account the complex sampling design of NHANES. The baseline tables were prepared using the “tableone” software package, while the data were processed and analyzed using the “survey” software package. The baseline table was first created to show the distribution of the overall characteristics of the participants, using chi‐square tests and *t‐*tests, and then further stratified according to cognitive functioning as assessed by cognitive tests. Choline intake was considered a categorical variable and divided into four quartiles. Continuous variables were represented by mean and standard deviation, whereas categorical variables were represented by sample size (*n*) and associated proportions. Sample size (*n*) was not affected by weighting adjustments, whereas proportions, means, and standard deviations were adjusted according to the weights. Subsequently, a weighted logistic regression model was developed to determine OR and 95% CI for the relationship between dietary choline intake and cognitive function.

Stepwise adjustments for potential confounders were made, and three hierarchical models were constructed: crude (unadjusted for confounders), model I (adjusted for age, race, and sex), and model II (adjusted for sex, age, race, education, marital status, PIR, BMI, smoking status, alcohol intake status, diabetes diagnosis, and hypertension diagnosis). In the above model, gender, age, and comorbidity (hypertension and diabetes) were analyzed in subgroups.

The RCS approach was used to investigate potential nonlinear correlations between dietary choline intake and cognitive function. Four nodes were positioned at the 5th, 35th, 65th, and 95th percentiles of the distribution of dietary choline intake to analyze the shape of the dose–response relationship between choline intake and risk of cognitive function. The RCS analysis did not take into account the complex sampling design of NHANES. In the dose–response relationship, we estimated Poverall and Pnonlinear values to assess statistical significance using the ANOVA function in the “rms” package. The values of Poverall and Pnonlinear were both less than 0.05, indicating the existence of a nonlinear dose–response relationship. If only Poverall is less than 0.05, it indicates a linear dose–response relationship.

## 3 Results

### 3.1 Baseline Characteristics of Study Participants

The study population's cognitive status characteristics are listed in Table 1. The sample used for the final multivariate regression analyses consisted of 2699 participants aged 60 years or older. In cognitive testing, individuals with low cognitive function exhibited significant differences from the general population in age distribution, ethnicity, PIR, educational attainment, total dietary energy intake, dietary choline intake, total choline intake, diabetes diagnosis, and hypertension diagnosis. In the CERAD‐WL test and DSST, participants with low cognitive function were mainly concentrated in the age range of ≥ 80 years, while in the CERAD‐DR test and AFT, participants with low cognitive function were mainly concentrated in the age range of 60–69 years old. Participants exhibiting low cognitive functioning on the CERAD test were predominantly male, whereas on the AFT & DSST, there were more females. Non‐Hispanic whites comprised 79% of the study population, and low cognitive function was also concentrated in this population. In the CERAD‐WL test, the overweight participants the poorer cognitive function. The majority of those who showed low cognitive function on the CERAD‐WL test, AFT, and DSST were in a state of ongoing alcohol use. High school and below education, married and cohabiting marital status, and low‐calorie and choline diets were all reflected in participants with low cognitive function in all tests.

**TABLE 1:** Characteristics of the study population, NHANES 2011–2014 cycles.

Characteristic	Overall		CERAD‐WL test			CERAD‐DR test			Animal fluency test			Digit symbol test		
	N^a^	Overall, *N* = 2699 (100%)^b^	Low Cognitive function, *N* = 753 (21%)^b^	Normal Cognitive function, *N* = 1946 (79%)^b^	p‐value^c^	Low Cognitive function, *N* = 1084 (35%)^b^	Normal Cognitive function, *N* = 1615 (65%)^b^	p‐value^c^	Low Cognitive function, *N* = 802 (21%)^b^	Normal Cognitive function, *N* = 1897 (79%)^b^	p‐value^c^	Low Cognitive function, *N* = 710 (16%)^b^	Normal Cognitive function, *N* = 1989 (84%)^b^	p‐values^c^
Age	2699				< 0.001			< 0.001			< 0.001			< 0.001
60–69 years		1364 (52%)	277 (30%)	1087 (58%)		422 (37%)	942 (61%)		331 (34%)	1033 (57%)		281 (31%)	1083 (56%)	
70–79 years		745 (27%)	226 (34%)	519 (25%)		320 (31%)	425 (25%)		237 (34%)	508 (25%)		213 (31%)	532 (26%)	
80+ years		590 (20%)	250 (35%)	340 (16%)		342 (32%)	248 (14%)		234 (32%)	356 (17%)		216 (38%)	374 (17%)	
Sex	2699				< 0.001			< 0.001			0.8			0.3
Female		1370 (54%)	298 (44%)	1072 (56%)		447 (45%)	923 (59%)		414 (55%)	956 (54%)		309 (51%)	1061 (54%)	
Male		1329 (46%)	455 (56%)	874 (44%)		637 (55%)	692 (41%)		388 (45%)	941 (46%)		401 (49%)	928 (46%)	
PIR	2495	3.10 (1.67, 5.00)	2.02 (1.16, 3.64)	3.48 (1.89, 5.00)	< 0.001	2.42 (1.35, 4.28)	3.48 (1.89, 5.00)	< 0.001	2.12 (1.20, 3.81)	3.40 (1.89, 5.00)	< 0.001	1.62 (1.01, 2.57)	3.48 (1.93, 5.00)	< 0.001
Race	2699				< 0.001			0.018			< 0.001			< 0.001
Non‐Hispanic White		1329 (79%)	338 (72%)	991 (81%)		515 (76%)	814 (80%)		282 (63%)	1047 (83%)		211 (55%)	1118 (83%)	
Non‐Hispanic Black		637 (8.5%)	175 (10.0%)	462 (8.1%)		275 (9.8%)	362 (7.8%)		268 (17%)	369 (6.2%)		243 (21%)	394 (6.2%)	
Other Hispanic		270 (3.8%)	101 (6.6%)	169 (3.0%)		124 (5.1%)	146 (3.1%)		96 (6.1%)	174 (3.1%)		134 (12%)	136 (2.2%)	
Mexican American		232 (3.5%)	80 (5.4%)	152 (3.0%)		106 (4.4%)	126 (3.1%)		62 (4.6%)	170 (3.2%)		89 (8.4%)	143 (2.6%)	
Other/multiracial		231 (5.3%)	59 (6.0%)	172 (5.1%)		64 (4.7%)	167 (5.6%)		94 (9.1%)	137 (4.3%)		33 (3.5%)	198 (5.6%)	
BMI	2664				0.010			0.12			0.3			0.3
Underweight (< 18.5)		38 (1.4%)	19 (3.1%)	19 (0.9%)		21 (1.9%)	17 (1.1%)		18 (2.2%)	20 (1.2%)		17 (2.6%)	21 (1.2%)	
Normal (18.5 to < 25)		676 (25%)	192 (26%)	484 (25%)		269 (25%)	407 (26%)		216 (28%)	460 (25%)		177 (27%)	499 (25%)	
Overweight (25 to < 30)		935 (36%)	277 (39%)	658 (35%)		400 (39%)	535 (34%)		268 (36%)	667 (36%)		240 (34%)	695 (36%)	
Obese (30 or greater)		1015 (38%)	249 (32%)	766 (39%)		377 (34%)	638 (39%)		280 (34%)	735 (38%)		255 (37%)	760 (38%)	
Alcohol drinking	2678				0.001			0.4			< 0.001			< 0.001
1–5 drinks/month		1301 (48%)	356 (43%)	945 (49%)		519 (48%)	782 (48%)		391 (47%)	910 (48%)		356 (47%)	945 (48%)	
5–10 drinks/month		120 (5.1%)	37 (5.8%)	83 (5.0%)		46 (4.9%)	74 (5.3%)		27 (2.9%)	93 (5.7%)		21 (2.3%)	99 (5.7%)	
10+ drinks/month		422 (20%)	97 (16%)	325 (21%)		162 (18%)	260 (21%)		78 (12%)	344 (22%)		61 (10%)	361 (22%)	
Non‐drinker		835 (27%)	250 (35%)	585 (25%)		343 (30%)	492 (26%)		298 (38%)	537 (24%)		260 (40%)	575 (25%)	
Smoking status	2699				0.5			0.6			0.3			0.2
Current smoker		340 (11%)	97 (10%)	243 (11%)		146 (10%)	194 (11%)		111 (12%)	229 (10%)		117 (13%)	223 (10%)	
Former smoker		1043 (40%)	285 (38%)	758 (40%)		434 (41%)	609 (39%)		295 (37%)	748 (40%)		258 (38%)	785 (40%)	
Never smoker		1316 (50%)	371 (52%)	945 (49%)		504 (48%)	812 (50%)		396 (50%)	920 (49%)		335 (49%)	981 (50%)	
Education	2699				< 0.001			< 0.001			< 0.001			< 0.001
High school or less		1305 (38%)	483 (57%)	822 (33%)		645 (50%)	660 (32%)		524 (59%)	781 (32%)		560 (73%)	745 (32%)	
Some college		768 (31%)	155 (24%)	613 (33%)		245 (25%)	523 (35%)		178 (25%)	590 (33%)		103 (18%)	665 (34%)	
College graduate or higher		626 (30%)	115 (19%)	511 (34%)		194 (25%)	432 (33%)		100 (16%)	526 (34%)		47 (9.4%)	579 (34%)	
Marital status	2699				< 0.001			0.072			0.011			< 0.001
Married/Living with partner		1573 (66%)	421 (59%)	1152 (67%)		610 (63%)	963 (67%)		433 (58%)	1140 (67%)		359 (51%)	1214 (68%)	
Widowed/Divorced/Separated		973 (30%)	290 (36%)	683 (28%)		416 (33%)	557 (28%)		329 (37%)	644 (28%)		312 (44%)	661 (28%)	
Never married		153 (4.3%)	42 (4.3%)	111 (4.3%)		58 (3.9%)	95 (4.5%)		40 (4.8%)	113 (4.2%)		39 (4.6%)	114 (4.2%)	
Total calories	2699	1794 (1412, 2252)	1700 (1273, 2129)	1812 (1453, 2276)	< 0.001	1756 (1320, 2211)	1807 (1463, 2263)	0.011	1609 (1254, 2106)	1835 (1463, 2276)	< 0.001	1528 (1143, 1940)	1845 (1469, 2287)	< 0.001
Dietary choline	2699	294 (221, 393)	270 (204, 360)	301 (227, 399)	< 0.001	277 (211, 374)	304 (227, 397)	0.006	266 (199, 360)	304 (228, 400)	< 0.001	260 (192, 351)	301 (226, 399)	< 0.001
Supplement choline	109	30 (1, 50)	50 (10, 100)	25 (1, 50)	0.3	40 (10, 100)	25 (0, 50)	0.3	17 (1, 100)	30 (1, 50)	0.8	100 (8, 110)	25 (1, 50)	0.2
Total choline	2699	297 (222, 395)	272 (204, 360)	305 (228, 400)	< 0.001	282 (211, 381)	307 (228, 399)	0.005	267 (199, 360)	307 (228, 401)	< 0.001	260 (192, 351)	305 (227, 400)	< 0.001
Hypertension	2699	1687 (58%)	486 (65%)	1201 (57%)	0.023	695 (63%)	992 (56%)	0.014	548 (67%)	1139 (56%)	< 0.001	491 (72%)	1196 (56%)	< 0.001
Diabetes	2699	627 (19%)	203 (23%)	424 (18%)	0.057	275 (23%)	352 (17%)	0.003	224 (26%)	403 (17%)	0.004	224 (31%)	403 (17%)	< 0.001

Abbreviations: BMI, body mass index; PIR, poverty impact ratio.

^a^ *N* not Missing (unweighted).

^b^ *n* (unweighted) (%); Median (Q1, Q3).

^c^ Pearson's X^2: Rao & Scott adjustment; Design‐based Kruskal–Wallis test.

### 3.2 Association Between Choline Intake and Cognitive Function

The results of the association analysis between choline intake and scores on two cognitive function tests (CERAD‐WL, CERAD‐DR) are shown in Tables 2 and 3, respectively. Multivariate logistic regression analysis revealed that in Model 1 (unadjusted for confounders), dietary choline and total choline intake were significantly associated with CERAD‐WL test scores. Specifically, each unit increase in choline intake was associated with a 36% reduction in the risk of adverse cognitive outcomes on the CERAD‐WL test (OR = 0.64, 95% CI = 0.50 ~ 0.81) and 35% (OR = 0.65, 95% CI = 0.51 ~ 0.82), respectively. For the CERAD‐DR test, analysis grouped by intake quartiles showed that before adjusting for confounders, each quartile increase in dietary choline and total choline intake was associated with a 24% reduction in the risk of adverse cognitive outcomes (OR = 0.76, 95% CI = 0.61 ~ 0.94) and 23% (OR = 0.77, 95% CI = 0.62 ~ 0.96), respectively. After adjusting for confounders, the positive association between choline intake and both cognitive function scores remained stable Table 2.

**TABLE 2:** Association of choline intake with CERAD‐WL test among participants in the NHANES 2011–2014 cycles. Model1: Unadjusted (crude model); Model2: Adjusted for sex, age, race; Model3: Adjusted for sex, age, race, education, marital status, PIR, alcohol drinking, smoking status, BMI, hypertension, diabetes.

	Model1		Model2		Model3	
	OR (95% CI)	*p*	OR (95% CI)	*p*	OR (95% CI)	*p*
Dietary choline						
Q1	1.00 (Reference)		1.00 (Reference)		1.00 (Reference)	
Q2	0.84 (0.67 ~ 1.05)	0.132	0.81 (0.64 ~ 1.03)	0.082	0.83 (0.64 ~ 1.08)	0.162
Q3	0.71 (0.57 ~ 0.90)	0.004	0.64 (0.50 ~ 0.82)	< 0.001	0.72 (0.55 ~ 0.94)	0.017
Q4	0.64 (0.50 ~ 0.81)	< 0.001	0.55 (0.42 ~ 0.71)	< 0.001	0.61 (0.46 ~ 0.81)	< 0.001
*p* for trend	< 0.001		< 0.001		0.003	
Total choline						
Q1	1.00 (Reference)		1.00 (Reference)		1.00 (Reference)	
Q2	0.85 (0.68 ~ 1.07)	0.173	0.82 (0.65 ~ 1.05)	0.111	0.84 (0.65 ~ 1.09)	0.192
Q3	0.71 (0.56 ~ 0.90)	0.004	0.65 (0.50 ~ 0.83)	< 0.001	0.72 (0.55 ~ 0.95)	0.019
Q4	0.65 (0.51 ~ 0.82)	< 0.001	0.56 (0.43 ~ 0.72)	< 0.001	0.62 (0.47 ~ 0.83)	0.001
*p* for trend	< 0.001		< 0.001		0.003	

Multivariate‐adjusted Model 3 analysis revealed that for CERAD‐WL test scores, dietary choline and total choline intake reduced the risk of adverse cognitive outcomes by 39% (OR = 0.61, 95% CI = 0.46 ~ 0.81) and 38% (OR = 0.62, 95% CI = 0.47 ~ 0.83) for total choline intake, respectively. For CERAD‐DR test scores, both reduced the risk of adverse cognitive outcomes by 34% (OR = 0.66, 95% CI = 0.51 ~ 0.85) and 32% (OR = 0.68, 95% CI = 0.53 ~ 0.88), respectively Table 3.

**TABLE 3:** Association of choline intake with CERAD‐DR test among participants in the NHANES 2011–2014 cycles. Model1: Unadjusted (crude model); Model2: Adjusted for sex, age, race; Model3: Adjusted for sex, age, race, education, marital status, PIR, alcohol drinking, smoking status, BMI, hypertension, diabetes.

	Model1		Model2		Model3	
	OR (95% CI)	*p*	OR (95% CI)	*p*	OR (95% CI)	*p*
Dietary choline						
Q1	1.00 (Reference)		1.00 (Reference)		1.00 (Reference)	
Q2	0.86 (0.70 ~ 1.06)	0.164	0.83 (0.67 ~ 1.04)	0.114	0.85 (0.66 ~ 1.08)	0.175
Q3	0.73 (0.59 ~ 0.90)	0.004	0.66 (0.52 ~ 0.83)	< 0.001	0.68 (0.53 ~ 0.87)	0.002
Q4	0.76 (0.61 ~ 0.94)	0.011	0.64 (0.51 ~ 0.82)	< 0.001	0.66 (0.51 ~ 0.85)	0.002
*p* for trend	0.014		< 0.001		0.003	
Total choline						
Q1	1.00 (Reference)		1.00 (Reference)		1.00 (Reference)	
Q2	0.88 (0.72 ~ 1.09)	0.246	0.86 (0.69 ~ 1.07)	0.183	0.87 (0.68 ~ 1.11)	0.265
Q3	0.71 (0.58 ~ 0.89)	0.002	0.65 (0.51 ~ 0.82)	< 0.001	0.66 (0.51 ~ 0.85)	0.001
Q4	0.77 (0.62 ~ 0.96)	0.019	0.67 (0.52 ~ 0.85)	< 0.001	0.68 (0.53 ~ 0.88)	0.004
*p* for trend	0.009		< 0.001		0.002	

Table 4 analyzes the association between choline intake and AFT. In unadjusted Model 1, higher choline intake levels were significantly associated with better AFT performance (dietary choline OR = 0.47, 95% CI = 0.37 ~ 0.60; total choline OR = 0.46, 95% CI = 0.36 ~ 0.58). In the fully multivariable‐adjusted model (Model 3), participants in the highest quartile of dietary choline and total choline intake had approximately 34% (OR = 0.66, 95% CI = 0.50 ~ 0.88) and 35% (OR = 0.65, 95% CI = 0.49 ~ 0.86), respectively, compared to the lowest quartile intake group.

**TABLE 4:** Association of choline intake with AFT among participants in the NHANES 2011–2014 cycles. Model1: Unadjusted (crude model); Model2: Adjusted for sex, age, race; Model3: Adjusted for sex, age, race, education, marital status, PIR, alcohol drinking, smoking status, BMI, hypertension, diabetes.

	Model1		Model2		Model3	
	OR (95% CI)	*p*	OR (95% CI)	*p*	OR (95% CI)	*p*
Dietary choline						
Q1	1.00 (Reference)		1.00 (Reference)		1.00 (Reference)	
Q2	0.73 (0.59 ~ 0.91)	0.005	0.76 (0.60 ~ 0.96)	0.020	0.83 (0.64 ~ 1.07)	0.152
Q3	0.58 (0.46 ~ 0.73)	< 0.001	0.64 (0.51 ~ 0.82)	< 0.001	0.74 (0.56 ~ 0.96)	0.025
Q4	0.47 (0.37 ~ 0.60)	< 0.001	0.53 (0.41 ~ 0.69)	< 0.001	0.66 (0.50 ~ 0.88)	0.004
*p* for trend	< 0.001		< 0.001		< 0.001	
Total choline						
Q1	1.00 (Reference)		1.00 (Reference)		1.00 (Reference)	
Q2	0.73 (0.59 ~ 0.91)	0.006	0.77 (0.61 ~ 0.96)	0.023	0.83 (0.64 ~ 1.07)	0.141
Q3	0.56 (0.44 ~ 0.70)	< 0.001	0.62 (0.49 ~ 0.79)	< 0.001	0.72 (0.55 ~ 0.94)	0.017
Q4	0.46 (0.36 ~ 0.58)	< 0.001	0.52 (0.40 ~ 0.67)	< 0.001	0.65 (0.49 ~ 0.86)	0.003
*p* for trend	< 0.001		< 0.001		< 0.001	

The results of the association analysis between dietary choline intake, total choline intake, and DSST scores are shown in Table 5. In Model 3, which adjusted for confounding factors, multivariate analysis grouped by intake quartiles showed that dietary choline intake (OR = 0.58, 95% CI = 0.42 ~ 0.80) and total choline intake (OR = 0.57, 95% CI = 0.42 ~ 0.79) remained significantly associated with DSST scores. For each quartile increase in intake, the risk of adverse cognitive outcomes decreased by 42% and 43%, respectively.

**TABLE 5:** Association of choline intake with DSST among participants in the NHANES 2011–2014 cycles. Model1: Unadjusted (crude model); Model2: Adjusted for sex, age, race; Model3: Adjusted for sex, age, race, education, marital status, PIR, alcohol drinking, smoking status, BMI, hypertension, diabetes.

	Model1		Model2		Model3	
	OR (95% CI)	*p*	OR (95% CI)	*p*	OR (95% CI)	*p*
Dietary choline						
Q1	1.00 (Reference)		1.00 (Reference)		1.00 (Reference)	
Q2	0.66 (0.52 ~ 0.83)	< 0.001	0.67 (0.52 ~ 0.86)	0.002	0.67 (0.50 ~ 0.90)	0.009
Q3	0.57 (0.45 ~ 0.72)	< 0.001	0.56 (0.43 ~ 0.73)	< 0.001	0.61 (0.45 ~ 0.83)	0.002
Q4	0.49 (0.39 ~ 0.63)	< 0.001	0.47 (0.35 ~ 0.62)	< 0.001	0.58 (0.42 ~ 0.80)	0.001
*p* for trend	< 0.001		< 0.001		0.006	
Total choline						
Q1	1.00 (Reference)		1.00 (Reference)		1.00 (Reference)	
Q2	0.68 (0.54 ~ 0.85)	< 0.001	0.68 (0.53 ~ 0.88)	0.003	0.68 (0.51 ~ 0.92)	0.011
Q3	0.54 (0.42 ~ 0.68)	< 0.001	0.54 (0.41 ~ 0.70)	< 0.001	0.58 (0.43 ~ 0.79)	< 0.001
Q4	0.49 (0.39 ~ 0.63)	< 0.001	0.47 (0.36 ~ 0.62)	< 0.001	0.57 (0.42 ~ 0.79)	< 0.001
*p* for trend	< 0.001		< 0.001		0.004	

Trend analyses showed that adjusting for candidate variables other than total dietary energy intake was associated with a progressively lower risk of low cognitive function as dietary choline intake increased in the final calibrated model (trend *p‐*values for the CERAD‐WL test, CERAD‐DR test, AFT, and DSST were 0.003, 0.003, < 0.001 and 0.006). Similarly, the total choline intake with inclusion of supplemental choline intake possessed a similar trend, showing a correlation with reduced risk of low cognitive function. (*p*‐values for trends in CERAD‐WL test, CERAD‐DR test, AFT, and DSST were 0.003, 0.002, < 0.001 and 0.004 respectively).

Multiple imputation indicated that a positive association between choline intake and cognitive test scores in elderly patients, demonstrating robust results (Tables S1, S2, S3, and S4).

### 3.3 Subgroup Analyses

**FIGURE 2:**
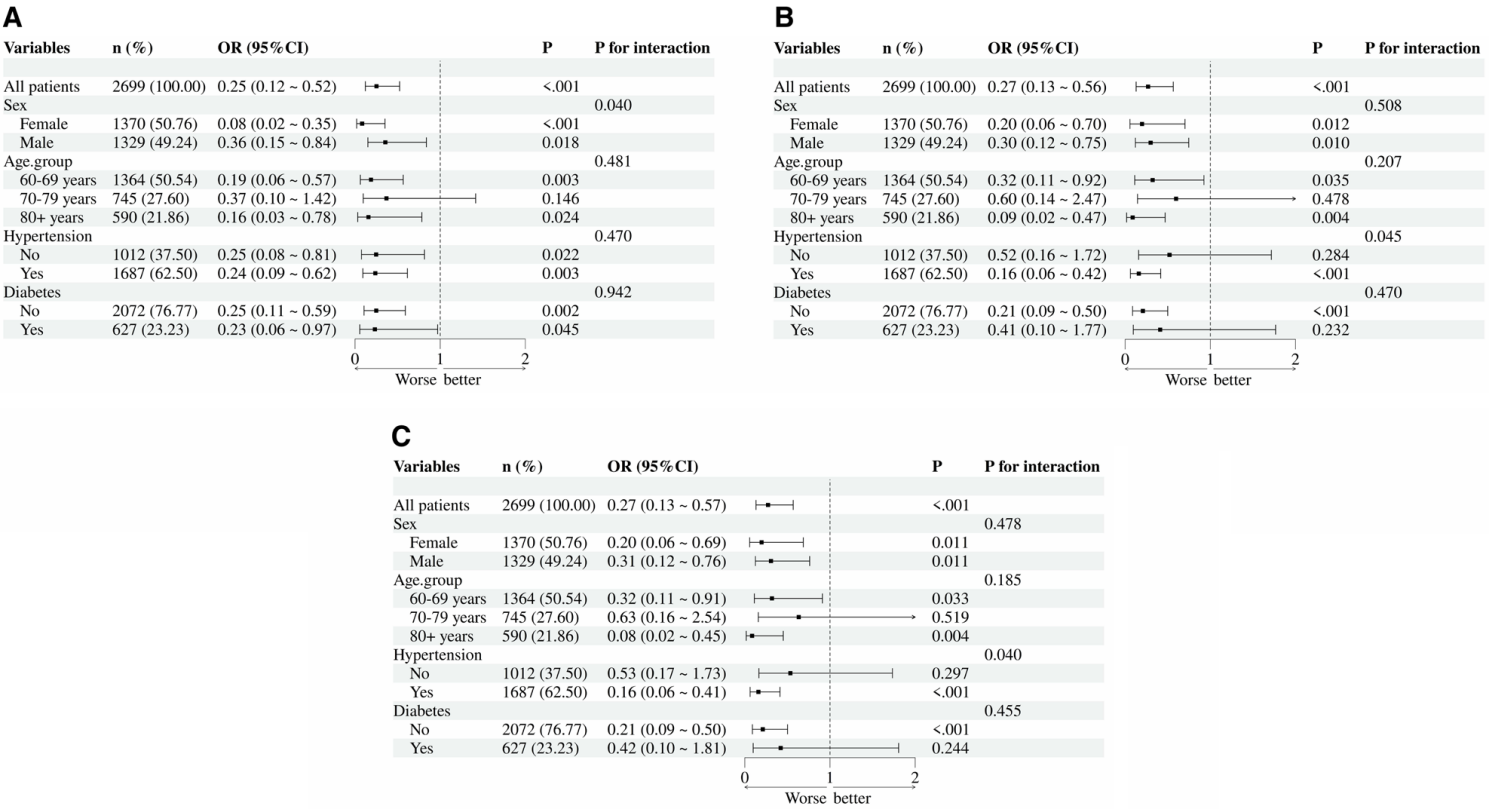
Subgroup analysis of choline intake and low cognitive function risk stratified by baseline characteristics. Corrected sex, age, race, education, marital status, PIR, alcohol drinking, smoking status, BMI, hypertension, diabetes. (A) Total choline with CERAD‐WL test, (B) dietary choline with AFT, (C) total choline with AFT.

Subgroup analysis evaluated whether there were differences in the association between choline intake and low cognitive function among subgroups (Figure 2). There was no significant difference in age and diabetes status (all *p* for interactions were > 0.05). However, a gender interaction was observed in the association between total choline intake and low cognitive function (CERAD‐WL test) (*p* = 0.040), with a stronger correlation observed in female patients (OR = 0.08, ‘95% CI’ = 0.02 ~ 0.35) compared to male patients (OR = 0.36, ‘95% CI’ = 0.15 ~ 0.84). In addition, the association between dietary choline/total choline intake and low cognitive function (AFT test) is influenced by the interaction of hypertension status (*p* = 0.045, 0.040), with stronger correlations observed in hypertensive patients (OR = 0.16, ‘95% CI’ = 0.06 ~ 0.42) and (OR = 0.16, ‘95% CI’ = 0.06 ~ 0.41). The remaining subgroup analysis results can be found in the supporting information.

### 3.4 Dose–Response Associations Between Choline Intake and Cognitive Function

**FIGURE 3:**
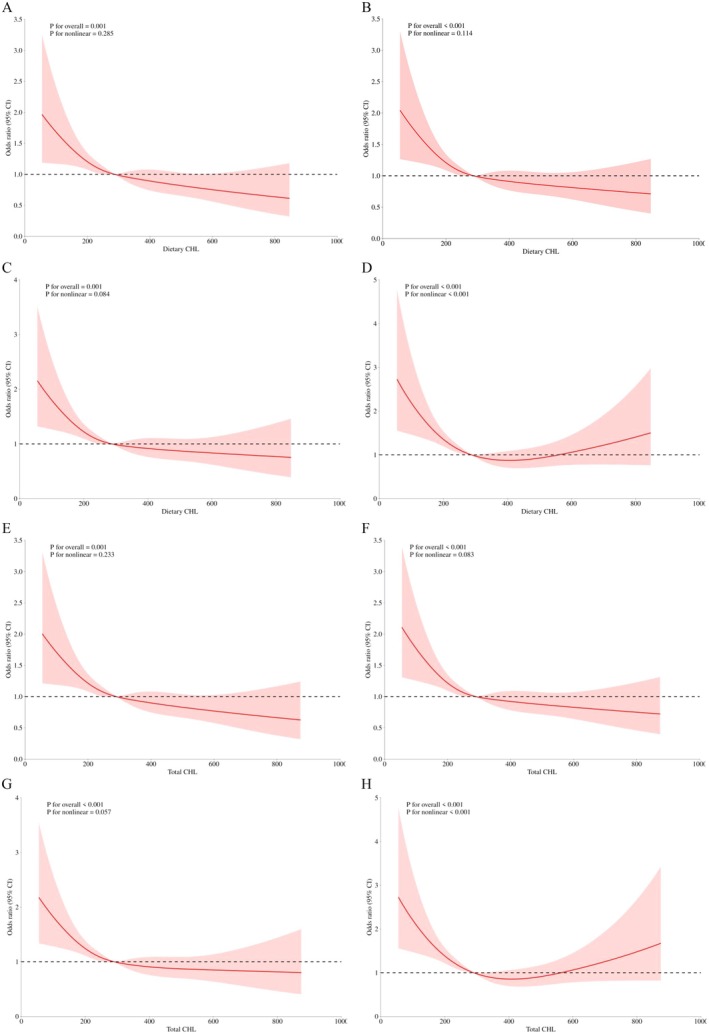
RCS analysis of dose–response relationships between choline intake and cognitive function. Adjusted for sociodemographic variables, BMI, alcohol drinking, smoking status, and comorbidities (hypertension, diabetes). CHL choline. (A, E) CERAD‐WL test; (B, F) CERAD‐DR test; (C, G) AFT; (D, H) DSST.

To further clarify the possible nonlinear relationship between choline intake and cognitive function, we performed a restricted cubic spline analysis based on Model 3 to assess the dose–response relationship of choline. The results showed that after adjusting for multifactors, Figure 3A–C did not observe significant nonlinear relationships between dietary choline intake and CERAD‐WL, CERAD‐DR, and AFT (Pnonlinear = 0.285, 0.114, and 0.084), and Figure 3D shows U‐shaped curves indicating a significant nonlinear correlation between dietary choline and DSST (Poverall < 0.001, Pnonline < 0.001).

Figure 3E–G similarly failed to show a significant nonlinear correlation between total choline intake and CERAD‐WL and CERAD‐DR, and AFT (Pnonlinear = 0.233, 0.083, and 0.057), and Figure 3H similarly showed a U‐shaped curve indicating a significant nonlinear correlation between total choline intake and DSST (Poverall < 0.001, Pnonlinear < 0.001).

## 4 Discussion

This study conducted a secondary analysis of a nationally representative sample of elderly adults to assess the association between choline intake and cognitive function. After adjusting for confounding factors, dietary choline intake and total choline intake were statistically associated with a reduced risk of cognitive impairment, as measured by cognitive test scores. Furthermore, interaction analyses indicated that gender and hypertension status may influence the association between choline intake and cognitive function. Dose–response analysis revealed a nonlinear relationship between choline intake and cognitive test scores.

Based on the hypothesis proposed by Poly et al. (Poly et al. 2011), adequate dietary choline intake may promote choline transport across the blood–brain barrier, thereby maintaining acetylcholine concentrations to ensure cholinergic neurotransmission. This suggests that an optimal level of choline intake may exist to mitigate age‐related cognitive decline.

Subsequently, two recent observational studies conducted in China have provided reference points for the effective dose of choline intake. In Guan's study, individuals in the high dietary total choline (> 205 mg/d) group and the high choline supplement (> 18.11 mg/d) intake group had higher overall cognitive scores (Guan et al. 2024). In Huang's study, the higher the intake of dietary choline (with an average maximum of 312.8 mg/d for males and 298.5 mg/d for females), the better the cognitive function of middle‐aged and elderly men and women in China (Huang et al. 2024). These also suggest that higher choline intake may yield greater cognitive benefits, though the reality is more complex.

In the study by Liu (Liu et al. 2021), a total choline intake of 187.6–399.5 mg/day was able to reduce the risk of low cognitive performance, including in terms of learning, categorical verbal fluency, working memory, and processing speed, and sustained attention. Additionally, Niu's cohort study revealed that moderate dietary choline intake (332.89 to 353.93 mg/day) was associated with a lower incidence of dementia and better cognitive function (Niu et al. 2025).

However, the aforementioned two studies rigidly categorized choline intake into quartiles, which may have resulted in information loss due to the discretization of a continuous variable, thereby reducing statistical power for detecting genuine associations. This study, utilizing RCS curves, not only confirmed the nonlinear relationship between choline intake and cognitive function but also provided a relatively reliable optimal intake range for the elderly. A significant U‐shaped correlation was observed between choline intake and DSST score, suggesting that moderate dietary choline intake (from 240.78 to 343.44 mg/day) and total choline intake (from 242.90 to 345.63 mg/day) are significantly associated with a reduced risk of cognitive impairment.

Based on Yuan et al.'s study (Yuan et al. 2022), it was found that high choline intake is associated with a higher risk of developing dementia and Alzheimer's disease, although these data are not statistically significant. We hypothesize that the failure of high choline intake to prevent the risk of cognitive impairment is due to the action of trimethylamine N‐oxide (TMAO) (Craciun and Balskus 2012; Zeisel and Warrier 2017). In the presence of intestinal bacteria, choline is metabolized into trimethylamine (TMA), which is then oxidized to TMAO. TMAO is currently known to be associated with mild cognitive impairment, AD dementia, and an increased risk of more severe AD pathology (Del Rio et al. 2017; Vogt et al. 2018). Recent studies have further confirmed that TMAO induces hippocampal‐dependent learning and memory impairment and synaptic plasticity defects by activating the mTOR signaling pathway (Zhou et al. 2023), which may be the reason for the gradual increase in choline intake leading to a decrease in DSST scores.

Our subgroup analysis showed that there was a significant interaction between choline intake and hypertension status. Compared with people without hypertension, there was a stronger negative correlation between choline intake and the incidence rate of cognitive impairment in hypertensive patients. Although the exact mechanism of this interaction is not fully understood, its impact on cerebral vascular structure is a possible explanation (Santisteban et al. 2023). Hypertension can directly affect cerebral small vessel remodeling (Rizzoni et al. 2009) and is associated with arterial stiffness, cerebral small vessel disease, cognitive decline, and dementia (van Sloten et al. 2015). In addition, extracranial atherosclerosis increases the risk of cognitive decline (Gardener et al. 2017). Hypertension promotes the formation and accumulation of carotid artery, vertebral artery, and intracranial cerebral artery atherosclerotic plaque (Hu et al. 2017; Iadecola and Gottesman 2019), which is due to the combination of shear stress and turbulence (Tabas et al. 2015), increased free radicals, and reduced nitric oxide signal transduction, accelerating inflammation and immune cell accumulation (Li et al. 2014). Choline, as a precursor for the synthesis of phosphatidylcholine, plays a crucial role in maintaining cell membrane integrity and reducing inflammation (Xu et al. 2024). Choline can inhibit phenotype transition, proliferation, migration, and vascular remodeling of vascular smooth muscle cells by activating M3AChR and Nrf2 antioxidant signaling pathways (He et al. 2020). It is also independently associated with the reduction of inflammatory mediators such as CRP, IL‐6, and TNF‐α (Detopoulou et al. 2008; Mazidi et al. 2019), which play a crucial role in cardiovascular disease. In addition, the impact of hypertension on neurodegenerative pathology also includes the deposition of amyloid‐β, which is the main component of amyloid plaques and a key pathological feature of AD (Santisteban et al. 2023). Supplementing choline is beneficial for patients with neurodegenerative diseases such as AD by increasing amyloid‐β, thioflavin S, and tau hyper‐phosphorylation (Eslami et al. 2024).

Another influencing factor in subgroup analysis is gender, which is similar to Huang's study (Huang et al. 2024), which emphasizes the neuroprotective benefits of choline in middle‐aged and elderly Chinese populations, especially in females. In Guan's study (Guan et al. 2024), it was also pointed out that the higher the total intake of choline, the more protective it is on cognitive ability, and the protective effect is more pronounced in the female population aged 55–65. Existing mechanistic evidence suggests that the neuroprotective effect of estrogen largely depends on its interaction with the cholinergic system. The liver can produce choline from phosphatidylethanolamine triple methylation to phosphatidylcholine (PC) through the action of phosphatidylethanolamine N‐methyltransferase (PEMT) gene (Fischer et al. 2007; Noga and Vance 2003). PEMT is significantly induced in response to estrogen, producing not only PC molecules and brain choline sources, but also a key source of long‐chain omega‐3 fatty acid DHA (Bortz et al. 2022). It is worth noting that 24% of European American women are homozygous for the PEMT rs12325817 effector allele, therefore requiring additional dietary choline to meet tissue choline needs (da Costa et al. 2014). This is also consistent with our research findings that low cognitive function is concentrated in the non‐Hispanic white population. Unfortunately, there is no data on estrogen in the NHANES database from 2011 to 2014, so further analysis cannot be conducted. It is necessary to conduct controlled feeding and supplementation trials on a series of doses of choline, carefully considering gender, menopausal status, and time, as well as PEMT genotype, to evaluate the potential role of choline in promoting neurocognition, especially in enhancing estrogen replacement.

This study has several noteworthy limitations. First, the NHANES database is cross‐sectional, which makes it impossible to show a causal association between choline intake and cognitive function. Following a diagnosis or course of therapy, patients with cognitive impairment may have altered their dietary practices, including their use of choline. To overcome this restriction, prospective cohort studies involving long‐term follow‐up and frequent evaluations of choline intake are required. Second, the data from this investigation relied heavily on participant‐recalled surveys, which may be subject to measurement error, such as omitted or misreported food intake. Although we averaged choline intake from two nonconsecutive 24‐h recalls to minimize the effect of day‐to‐day variability, this method may not fully capture long‐term habitual intake. Once more, there are many possible causes of cognitive impairment. Despite these limitations, our study demonstrates a nonlinear relationship between choline intake and cognitive function scores.

## 5 Conclusion

We found that after adjusting for potential confounding factors, choline intake may reduce the risk of cognitive impairment to some extent, exhibiting a nonlinear relationship. This conclusion was validated through multiple sensitivity analyses. Future prospective cohort studies with larger sample sizes and repeated assessments of choline intake are needed to confirm these results and explore potential mechanisms. Given the global increase in the prevalence of cognitive impairment and dementia, this hypothesis may pave the way for future interventional studies to determine the effect of choline intake on cognitive function.

## Author Contributions

Yu‐hang Chen: writing – review and editing, writing – original draft, visualization, validation, supervision, software, resources, project administration, methodology, investigation, formal analysis, data curation, conceptualization. Ting‐ting Liu: writing – review and editing, supervision, methodology, formal analysis. Li‐ming Chen: writing – review and editing, supervision, methodology, conceptualization.

## Funding

The authors declare that no financial support was received for the research, authorship, and/or publication of this article.

## Ethics Statement

The NHANES surveys got informed consent from all original participants at the time of data collection and preserved participant confidentiality by anonymizing information before public release. This study was deemed exempt from the institutional review board of Chongqing Mental Health Center since it utilized the analysis of pre‐existing de‐identified data from the publicly accessible NHANES database.

## Conflicts of Interest

The authors declare no conflicts of interest.

## Supporting information


**Table S1:** Association of choline intake with CERAD‐WL test among participants in the NHANES 2011–2014 cycles. Model1: unadjusted (crude model); Model2: adjusted for Sex, Age, Race; Model3: adjusted for Sex, Age, Race, Education, Marital status, PIR, Alcohol drinking, Smoking status, BMI, Hypertension, Diabetes.
**Table S2:** Association of choline intake with CERAD‐DR test among participants in the NHANES 2011–2014 cycles. model1: unadjusted (crude model); Model2: adjusted for Sex, Age, Race; Model3: adjusted for Sex, Age, Race, Education, Marital status, PIR, Alcohol drinking, Smoking status, BMI, Hypertension, Diabetes.
**Table S3:** Association of choline intake with AFT among participants in the NHANES 2011–2014 cycles. Model1: unadjusted (crude model); Model2: adjusted for Sex, Age, Race; Model3: adjusted for Sex, Age, Race, Education, Marital status, PIR, Alcohol drinking, Smoking status, BMI, Hypertension, Diabetes.
**Table S4:** Association of choline intake with DSST among participants in the NHANES 2011–2014 cycles. Model1: unadjusted (crude model); Model2: adjusted for Sex, Age, Race; Model3: adjusted for Sex, Age, Race, Education, Marital status, PIR, Alcohol drinking, Smoking status, BMI, Hypertension, Diabetes.

## Data Availability

The datasets analyzed for this study are available via the CDC NHANES website (https://www.cdc.gov/nchs/nhanes).
