# Supplementary material for: Relationship Between Dietary Choline Intake and Cognitive Function in the United States: A Cross‐Sectional Study of the 2011–2014 NHANES Cycle
Source: Food Sci Nutr. 2026 Mar 11;14(3):e71631. doi: 10.1002/fsn3.71631 (PMC13093565; doi:10.1002/fsn3.71631)
Supplement: Supplementary file 1 — Table S1: Association of choline intake with CERAD‐WL test among participants in the NHANES 2011–2014 cycles. Model1: unadjusted (crude model); Model2: adjusted for Sex, Age, Race; Model3: adjusted for Sex, Age, Race, Education, Marital status, PIR, Alcohol drinking, Smoking status, BMI, Hypertension, Diabetes. Table S2: Association of choline intake with CERAD‐DR test among participants in the NHANES 2011–2014 cycles. model1: unadjusted (crude model); Model2: adjusted for Sex, Age, Race; Model3: adjusted for Sex, Age, Race, Education, Marital status, PIR, Alcohol drinking, Smoking status, BMI, Hypertension, Diabetes. Table S3: Association of choline intake with AFT among participants in the NHANES 2011–2014 cycles. Model1: unadjusted (crude model); Model2: adjusted for Sex, Age, Race; Model3: adjusted for Sex, Age, Race, Education, Marital status, PIR, Alcohol drinking, Smoking status, BMI, Hypertension, Diabetes. Table S4: Association of choline intake with DSST among participants in the NHANES 2011–2014 cycles. Model1: unadjusted (crude model); Model2: adjusted for Sex, Age, Race; Model3: adjusted for Sex, Age, Race, Education, Marital status, PIR, Alcohol drinking, Smoking status, BMI, Hypertension, Diabetes. [file FSN3-14-e71631-s001.docx]

Supplementary Material

|  | Model1 | |  | Model2 | |  | Model3 | | |
| --- | --- | --- | --- | --- | --- | --- | --- | --- | --- |
|  | OR (95% CI) | *P* |  | OR (95% CI) | *P* |  | OR (95% CI) | *P* | |
| Dietary choline |  |  |  |  |  |  |  |  | |
| Q1 | 1.00 (Reference) |  |  | 1.00 (Reference) |  |  | 1.00 (Reference) |  | |
| Q2 | 0.84 (0.67 ~ 1.05) | 0.132 |  | 0.81 (0.64 ~ 1.03) | 0.082 |  | 0.84 (0.66 ~ 1.07) | 0.164 | |
| Q3 | 0.71 (0.57 ~ 0.90) | **0.004** |  | 0.64 (0.50 ~ 0.82) | **<0.001** |  | 0.70 (0.54 ~ 0.91) | **0.008** | |
| Q4 | 0.64 (0.50 ~ 0.81) | **<0.001** |  | 0.55 (0.42 ~ 0.71) | **<0.001** |  | 0.64 (0.49 ~ 0.84) | **0.001** | |
| *P* for trend | **0.014** |  |  | **<0.001** |  |  | **0.015** |  | |
| Total choline |  |  |  |  |  |  |  |  | |
| Q1 | 1.00 (Reference) |  |  | 1.00 (Reference) |  |  | 1.00 (Reference) |  | |
| Q2 | 0.85 (0.68 ~ 1.07) | 0.173 |  | 0.82 (0.65 ~ 1.05) | 0.111 |  | 0.85 (0.67 ~ 1.09) | 0.197 | |
| Q3 | 0.71 (0.56 ~ 0.90) | **0.004** |  | 0.65 (0.50 ~ 0.83) | **<0.001** |  | 0.72 (0.56 ~ 0.93) | **0.013** | |
| Q4 | 0.65 (0.51 ~ 0.82) | **<0.001** |  | 0.56 (0.43 ~ 0.72) | **<0.001** |  | 0.65 (0.50 ~ 0.85) | **0.002** | |
| *P* for trend | **0.009** |  |  | **<0.001** |  |  | **0.012** |  | |
| Supplementary Tables S1 Association of choline intake with CERAD-WL test among participants in the NHANES 2011-2014 cycles. Model1: unadjusted (crude model); Model2: adjusted for Sex, Age, Race; Model3: adjusted for Sex, Age, Race, Education, Marital status, PIR, Alcohol drinking, Smoking status, BMI, Hypertension, Diabetes. | | | | | | | | |  |

|  | Model1 | |  | Model2 | |  | Model3 | |
| --- | --- | --- | --- | --- | --- | --- | --- | --- |
|  | OR (95% CI) | *P* |  | OR (95% CI) | *P* |  | OR (95% CI) | *P* |
| Dietary choline |  |  |  |  |  |  |  |  |
| Q1 | 1.00 (Reference) |  |  | 1.00 (Reference) |  |  | 1.00 (Reference) |  |
| Q2 | 0.86 (0.70 ~ 1.06) | 0.164 |  | 0.83 (0.67 ~ 1.04) | 0.114 |  | 0.86 (0.69 ~ 1.08) | 0.203 |
| Q3 | 0.73 (0.59 ~ 0.90) | **0.004** |  | 0.66 (0.52 ~ 0.83) | **<0.001** |  | 0.71 (0.56 ~ 0.90) | **0.005** |
| Q4 | 0.76 (0.61 ~ 0.94) | **0.011** |  | 0.64 (0.51 ~ 0.82) | **<0.001** |  | 0.72 (0.56 ~ 0.92) | **0.009** |
| *P* for trend | **<0.001** |  |  | **<0.001** |  |  | **<0.001** |  |
| Total choline |  |  |  |  |  |  |  |  |
| Q1 | 1.00 (Reference) |  |  | 1.00 (Reference) |  |  | 1.00 (Reference) |  |
| Q2 | 0.88 (0.72 ~ 1.09) | 0.246 |  | 0.86 (0.69 ~ 1.07) | 0.183 |  | 0.89 (0.70 ~ 1.11) | 0.294 |
| Q3 | 0.71 (0.58 ~ 0.89) | **0.002** |  | 0.65 (0.51 ~ 0.82) | **<0.001** |  | 0.71 (0.56 ~ 0.90) | **0.005** |
| Q4 | 0.77 (0.62 ~ 0.96) | **0.019** |  | 0.67 (0.52 ~ 0.85) | **<0.001** |  | 0.74 (0.58 ~ 0.95) | **0.018** |
| *P* for trend | **<0.001** |  |  | **<0.001** |  |  | **<0.001** |  |
| Supplementary Tables S2 Association of choline intake with CERAD-DR test among participants in the NHANES 2011-2014 cycles. model1: unadjusted (crude model); Model2: adjusted for Sex, Age, Race; Model3: adjusted for Sex, Age, Race, Education, Marital status, PIR, Alcohol drinking, Smoking status, BMI, Hypertension, Diabetes. | | | | | | | | |

|  | Model1 | |  | Model2 | |  | Model3 | |
| --- | --- | --- | --- | --- | --- | --- | --- | --- |
|  | OR (95% CI) | *P* |  | OR (95% CI) | *P* |  | OR (95% CI) | *P* |
| Dietary choline |  |  |  |  |  |  |  |  |
| Q1 | 1.00 (Reference) |  |  | 1.00 (Reference) |  |  | 1.00 (Reference) |  |
| Q2 | 0.73 (0.59 ~ 0.91) | **0.005** |  | 0.76 (0.60 ~ 0.96) | **0.020** |  | 0.80 (0.63 ~ 1.02) | 0.069 |
| Q3 | 0.58 (0.46 ~ 0.73) | **<0.001** |  | 0.64 (0.51 ~ 0.82) | **<0.001** |  | 0.71 (0.56 ~ 0.92) | **0.008** |
| Q4 | 0.47 (0.37 ~ 0.60) | **<0.001** |  | 0.53 (0.41 ~ 0.69) | **<0.001** |  | 0.64 (0.49 ~ 0.84) | **0.001** |
| *P* for trend | **<0.001** |  |  | **<0.001** |  |  | **<0.001** |  |
| Total choline |  |  |  |  |  |  |  |  |
| Q1 | 1.00 (Reference) |  |  | 1.00 (Reference) |  |  | 1.00 (Reference) |  |
| Q2 | 0.73 (0.59 ~ 0.91) | **0.006** |  | 0.77 (0.61 ~ 0.96) | **0.023** |  | 0.80 (0.63 ~ 1.02) | 0.070 |
| Q3 | 0.56 (0.44 ~ 0.70) | **<0.001** |  | 0.62 (0.49 ~ 0.79) | **<0.001** |  | 0.70 (0.54 ~ 0.90) | **0.005** |
| Q4 | 0.46 (0.36 ~ 0.58) | **<0.001** |  | 0.52 (0.40 ~ 0.67) | **<0.001** |  | 0.62 (0.48 ~ 0.81) | **<0.001** |
| *P* for trend | **<0.001** |  |  | **<0.001** |  |  | **<0.001** |  |
| Supplementary Tables S3 Association of choline intake with AFT among participants in the NHANES 2011-2014 cycles. Model1: unadjusted (crude model); Model2: adjusted for Sex, Age, Race; Model3: adjusted for Sex, Age, Race, Education, Marital status, PIR, Alcohol drinking, Smoking status, BMI, Hypertension, Diabetes. | | | | | | | | |

|  | Model1 | |  | Model2 | |  | Model3 | |
| --- | --- | --- | --- | --- | --- | --- | --- | --- |
|  | OR (95% CI) | *P* |  | OR (95% CI) | *P* |  | OR (95% CI) | *P* |
| Dietary choline |  |  |  |  |  |  |  |  |
| Q1 | 1.00 (Reference) |  |  | 1.00 (Reference) |  |  | 1.00 (Reference) |  |
| Q2 | 0.66 (0.52 ~ 0.83) | **<0.001** |  | 0.67 (0.52 ~ 0.86) | **0.002** |  | 0.71 (0.54 ~ 0.93) | **0.014** |
| Q3 | 0.57 (0.45 ~ 0.72) | **<0.001** |  | 0.56 (0.43 ~ 0.73) | **<0.001** |  | 0.67 (0.50 ~ 0.89) | **0.006** |
| Q4 | 0.49 (0.39 ~ 0.63) | **<0.001** |  | 0.47 (0.35 ~ 0.62) | **<0.001** |  | 0.62 (0.45 ~ 0.84) | **0.002** |
| *P* for trend | **<0.001** |  |  | **<0.001** |  |  | **0.022** |  |
| Total choline |  |  |  |  |  |  |  |  |
| Q1 | 1.00 (Reference) |  |  | 1.00 (Reference) |  |  | 1.00 (Reference) |  |
| Q2 | 0.68 (0.54 ~ 0.85) | **<0.001** |  | 0.68 (0.53 ~ 0.88) | **0.003** |  | 0.71 (0.54 ~ 0.94) | **0.017** |
| Q3 | 0.54 (0.42 ~ 0.68) | **<0.001** |  | 0.54 (0.41 ~ 0.70) | **<0.001** |  | 0.65 (0.48 ~ 0.86) | **0.003** |
| Q4 | 0.49 (0.39 ~ 0.63) | **<0.001** |  | 0.47 (0.36 ~ 0.62) | **<0.001** |  | 0.62 (0.45 ~ 0.84) | **0.002** |
| *P* for trend | **<0.001** |  |  | **<0.001** |  |  | **0.014** |  |
| Supplementary Tables S4 Association of choline intake with DSST among participants in the NHANES 2011-2014 cycles. Model1: unadjusted (crude model); Model2: adjusted for Sex, Age, Race; Model3: adjusted for Sex, Age, Race, Education, Marital status, PIR, Alcohol drinking, Smoking status, BMI, Hypertension, Diabetes. | | | | | | | | |
